# Big Data’s Role in Precision Public Health

**DOI:** 10.3389/fpubh.2018.00068

**Published:** 2018-03-07

**Authors:** Shawn Dolley

**Affiliations:** ^1^Cloudera, Inc., Palo Alto, CA, United States

**Keywords:** precision public health, big data, computational epidemiology, infectious disease surveillance, precision population health

## Abstract

Precision public health is an emerging practice to more granularly predict and understand public health risks and customize treatments for more specific and homogeneous subpopulations, often using new data, technologies, and methods. Big data is one element that has consistently helped to achieve these goals, through its ability to deliver to practitioners a volume and variety of structured or unstructured data not previously possible. Big data has enabled more widespread and specific research and trials of stratifying and segmenting populations at risk for a variety of health problems. Examples of success using big data are surveyed in surveillance and signal detection, predicting future risk, targeted interventions, and understanding disease. Using novel big data or big data approaches has risks that remain to be resolved. The continued growth in volume and variety of available data, decreased costs of data capture, and emerging computational methods mean big data success will likely be a required pillar of precision public health into the future. This review article aims to identify the precision public health use cases where big data has added value, identify classes of value that big data may bring, and outline the risks inherent in using big data in precision public health efforts.

## Introduction

This review article aims to identify the precision public health use cases where big data has added value, identify classes of value that big data may bring, and outline the risks inherent in using big data in precision public health efforts. This article focuses on surveying current practice, with a breadth of examples. The article does not include a critical review of the methods included in the big data and precision public health published research. It is hoped this article may pave the way for future researchers to measure the strengths and weaknesses, robustness, and validity of individual studies, interventions and outcomes. With the breadth of practice defined here, such follow-on in-depth critical review could identify precision public health best practices in design, methods, implementation, and analysis.

## Methods

The terms “big data” and “precision public health”—two relatively new disciplines—often do not appear in the nomenclature of contemporary public health interventions and studies. Searching for the terms “big data” or “precision public health” returns a small fraction of the actual activity. Based on the lack of existing reviews and the complexity in identifying the intersection of precision public health and big data, the rationale of this narrative review article is to find examples of the use of big data in implementations of precision public health published in peer-reviewed academic journals. The author (a) reviewed a large number of public health studies to look for precision and big data, as well as related and follow-on studies, (b) identified and searched for specific types of big data being applied to public health, and (c) searched for uses of data in precision public health to identify big vs. small data—always using the definition of these terms rather than relying on the presence of the terms “big data” or “precision public health.”

Searches were performed using Google Scholar and Google. Examples of public health implementations—with and without big data—and precision public health implementations—with and without big data—only qualified for this article if they were published in peer-reviewed journals. In the presence of multiple qualifying examples, best attempts were made to limit examples to a single citation. In the presence of multiple examples, to reduce risk of bias and attempt to identify the most robust examples, the examples selected were those with the (a) most clearly identifiable public health use case, (b) clearest use of big data, (c) most “precision,” (d) in journals with the highest impact factor, that were (e) the most recent—and in that order of priority. Searches were concluded by July 20, 2017.

Search terms used were as follows:

1. For identifying implementations using big data volume, the term “public health” and each of the following: “big data,” “gene-wide,” “genome,” “genomic,” “germline,” “GWAS,” “imaging,” “molecular,” “multi-omic,” “pan-omic,” “phenome,” “PWAS,” “translational,” “video,” “whole exome,” and “whole genome.”
2. For identifying implementations using big data variety, the term “public health” and each of the following: “big data,” “drone,” “Facebook,” “Instagram,” “IoT,” “internet of things,” “linked,” “linked data,” “patient-centered,” “patient generated,” “mobile,” “mobile phone,” “registry,” “registries,” “secondary use,” “semantic,” “sensors,” “social media,” “surveys,” “Twitter,” “UAV,” “unmanned aerial vehicle,” “variety,” and “wearable.”
3. For identifying implementations using big data velocity, the term “public health” and each of the following: “big data,” “continuous,” “monitor,” “real-time,” “sensor,” “streams,” “streaming,” “velocity,” and “video.”
4. For identifying public health implementations—including programs, trials, innovations and experiments—using big data, the term “big data” and each of the following: “adverse drug event,” “ADE,” “adverse event,” “cohort,” “epidemic,” “epidemiology,” “health intervention,” “health risk,” “heterogeneous,” “homogeneous,” “human movement,” “outcomes,” “pandemic,” “pharmaco-epidemiology,” “population health,” “precision public health,” “prevention,” “public health,” “signal detection,” “surveillance,” “targeted intervention,” “tracking,” “vaccine,” “vector,” and “virus.”

Google Scholar also provides lists of more recent studies which have cited the current study. These lists were reviewed to identify if more recent studies existed that provided better examples of pertinent characteristics.

This method has a number of limitations. Google Scholar has limitations, including relying on the end user to discriminate which studies returned are from peer-reviewed journals. No review protocol exists independent of this review article. No study selection or summary measures were collected, and no meta-analysis was performed. No study characteristics were collected. No assessment of the validity of included studies was performed beyond their inclusion in peer-reviewed academic journals. No assessment of cumulative level bias risk was performed. No additional analysis methods were used. The selection of studies included was not independently reviewed. The scope of this narrative review precludes enumerating additional limitations. Limitations aside, the result of these methods is a collection of studies or programs where big data and precision public health—as these terms are defined in this article—are being used together. Through implementing these methods, this review article is the first to identify the scope and scale of big data’s role in precision public health, highlight classes of innovation, and identify the risks of using big data in this field.

## Precision Public Health

“Precision public health is a new field driven by technological advances that enable more precise descriptions and analyzes of individuals and population groups, with a view to improving the overall health of populations” (1). The term was coined in Australia by Dr. Tarun Weeramanthri in 2013, and first found in print in 2014 (2). Dr. Muin Khoury and Dr. Sandro Galea describe precision public health as “improving the ability to prevent disease, promote health, and reduce health disparities in populations by applying emerging methods and technologies for measuring disease, pathogens, exposures, behaviors, and susceptibility in populations; and developing policies and targeted implementation programs to improve health” (3). Precision public health leverages big data and its enabling technologies to achieve a previously impossible level of targeting or speed (4). The Bill & Melinda Gates Foundation adds that precision public health “requires robust primary surveillance data, rapid application of sophisticated analytics to track the geographical distribution of disease, and the capacity to act on such information” (5). Precision public health works because “more-accurate methods for measuring disease, pathogens, exposures, behaviors, and susceptibility could allow better assessment of population health and development of policies and targeted programs for preventing disease” (4). Arnett & Claas add “Precision public health is characterized by discovering, validating, and optimizing care strategies for well-characterized population strata” (6). As for the size of the strata, Colijn et al. state “precision approaches must act at the right scale, which will often be intermediate—between “one size fits all” medicine and fully individualized therapies” (7).

The prominence of the term “precision” in the new practices of precision medicine and precision public health will invariably raise questions about their similarity. While precision medicine requires genetic, lifestyle, and environmental data to meet goals of more customized and potentially individualized clinical treatments, precision public health is about increased accuracy and granularity in defining public cohorts and delivering target interventions of many types (4–6). Precision medicine and precision public health are independent.

## Big Data in Healthcare and Public Health

Big data has recently become a ubiquitous approach to driving insights, innovation and new interventions across economic sectors (8, 9). The United States National Institute of Standards and Technology defines big data as follows: “Big Data consists of extensive datasets—primarily in the characteristics of volume, variety, velocity, and/or variability—that require a scalable architecture for efficient storage, manipulation, and analysis,” (10). Decreases in costs of technology enabled the big data phenomenon to emerge (11). Data of “such a high volume, velocity and variety to require specific technology and analytical methods for its transformation into value” has a symbiotic relationship with the technology innovation on which it relies; the term big data often conflates the actual physical data with the unique technologies required to use it (12, 13).

In patient-specific healthcare, big data technology has helped enable greater scales of volume, variety and velocity (14, 15). Usable data *volume* has significantly increased in areas such as genomics (16, 17), molecular research (18, 19), medical image mining (20), and population health (21, 22). Enabling a *variety* of data to be integrated, for a more complete view of patient or population, has occurred in areas including air quality (23, 24), wearables (25, 26), patient generated content *via* the web (27), patient or physician movement (28, 29), medical studies (30), and critical care (31). Big data enabling increased *velocity* in healthcare was one of the earliest uses, in areas such as clinical prediction (32, 33), and diagnostics (15, 33). Current examples and future vision for use of big data exists in multiple and varying pathologies, including cancer (34), cardiology (35), epilepsy (36), family medicine (37), gastroenterology (38), nursing (39), pediatric ophthalmology (40), psychiatry (41, 42), and women’s health (43) as examples.

Barrett et al. state succinctly: “Big data can play a key role in both research and intervention activities and accelerate progress in disease prevention and population health” (44). Big data shows utility across the entire spectrum of public health disciplines. This capability ranges from “monitoring population health in real-time” to building “definitive extents and databases on the occurrence of many diseases” (45). Public health subject areas that include examples of the use of big data include community health (46), environmental health science (24, 47), epidemiology (48), infectious disease (45), maternal and child health (49), occupational health and safety (50), and nutrition (51). There is optimism and evidence for big data’s value in public health, both in research and in intervention (52).

## Big Data in Precision Public Health

Today, use of big data has been shown to improve precision in select disciplines of public health. These areas include performing disease surveillance and signal detection (53, 54), predicting risk (55, 56), targeting interventions (6), and understanding disease (57). Research and proofs-of-concept with this data for these applications have been performed around the world. With the pace of technology innovation, and the speed at which precision health practitioners have embraced big data, there will likely be more public health disciplines, practices, approaches, and interventions implemented in the future or that are beyond the scope of this article (58, 59).

## Performing Disease Surveillance and Signal Detection

Disease surveillance and signal detection are among the most commonly cited and revolutionary of the big data use cases in precision public health (45, 60–62). Precision signal detection or disease surveillance using big data has shown efficacy in air pollution (23, 24), antibiotic resistance (63), cholera (64), dengue (65, 66), drowning (67), drug safety (68, 69), electromagnetic field exposure (70), Influenza A H1N1 (71), Lyme disease (72), monitoring food intake (73), and whooping cough (74).

Disease surveillance often includes tracking affected individuals, i.e., human carriers, patients, or victims (75). Stoddard et al. stated in 2009: “Human movement is a critical, understudied behavioral component underlying the transmission dynamics of many vector-borne pathogens” (76). In the effort to track disease spread by human vectors, a premium is placed on information that is more recent and granular (77, 78). Thus, access to huge volumes of streaming real-time data generated by humans seems at once an ideal signal repository for identifying and tracking affected individuals, and definitionally big data (78).

Indeed, big data supports alternate and in some ways superior methods to track affected individuals (45, 62). Because affected individuals move so quickly and at such a wide range, the real-time capabilities of big data and big data technology are now critical in this discipline (79, 80). Studies have shown efficacy using mobile phone data in tracking movement in cholera (81), dengue (82), Ebola (83), human immunodeficiency virus (HIV) (84), malaria (85), rubella (85), and schistosomiasis (86). Other mechanisms that have shown efficacy or promise in tracking movement of affected individuals include air travel data (87), GPS data-loggers (88), magnetometers (89), Twitter (71), and web searches (65).

## Predicting Risk

Effective signal detection often leads to attempts to predict future signals (90, 91). Predicting public health risk leads to a chance to implement preventive interventions (56, 92). Models predicting either disease spread or outcomes, using traditional or non-big data sources, have been developed across the spectrum of public health crises, including dengue (93), HIV (94), influenza (95), malaria (96), Rift Valley Fever (97), and tuberculosis (98).

One early example of using big data for public health prediction, Google Flu Trends, was a well-publicized failure (99). Since that episode, approaches to predicting risk using the internet and social media have shown special care to include merging big data with non-social media data sources, avoid overfitting models with relatively few cases, and being conscious of the risks of big data (56, 100).

Big data has been used for risk prediction of spread or outcomes in public health topics such as air pollution (101), antibiotic resistance (102), avian influenza A (103), blood lead levels (104), child abuse (49), diabetes (105), Ebola (106), HIV (107), malaria (108), gestational diabetes (109), smoking progression (110), West Nile (111), and Zika (86, 112, 113).

## Targeting Treatment Interventions

Applying treatment interventions to homogeneous cohorts within a larger heterogeneous population has been advocated since Lalonde’s seminal report “A New Perspective on the Health of Canadians” in 1974 (114). Historical examples of adding precision to public health treatment populations include gonorrhea in the 1980s (115), HIV in the 1990s (116), breast cancer in the 2000s (117), and malaria in the 2010s (118). In 2010, the US Department of Health and Human Services said of those citizens with multiple chronic conditions: “Indeed, developing means for determining homogeneous subgroups among this heterogeneous population is viewed as an important step in the effort to improve the health status of the total population” (119).

Big data was leveraged in public health research identifying finer-grain treatment interventions in childhood asthma (120), childhood obesity (121), diarrhea (122), Hepatitis C (123), HIV (124), injectable drug use (125), malaria (126), opioid medication misuse (127), use of smokeless tobacco (128), and the Zika virus (129).

One clinical example at the intersection of identifying subpopulations for effective interventions and big data is personalized vaccinology or “vaccinomics” (130). Most vaccines today are applied in a one-size fits all model: the typical implementation assumes a homogenous population, uses the same vaccine and dosages for all patients, ignores replicated, empirical realities of a heterogeneous population, and does not use sophisticated genomic capabilities at hand (131, 132). While today’s vaccines are applied homogeneously, the results are individual: “The response to a vaccine is the cumulative result of non-random interactions with host genes, epigenetic phenomena, metagenomics and the microbiome, gene dominance, complementarity, epistasis, coinfections, and other factors” (133). Vaccinomics would focus on homogeneous subpopulations treated with vaccines, dosages and approaches that would “hold the promise of moving away from one standard vaccine against all human populations…to one where vaccines can be relatively easily tailor-fitted to individual, community and population specificity” (134).

## Understanding Disease

Data volume and variety in epidemiology have grown consistently over time well before the age of big data (135–137). Contemporary exponential increases in data sizes, and perhaps more importantly increases in variety of data sources, make big data a valuable addition to the epidemiologist’s toolkit (64, 138). Glymour states “We recommend that social epidemiologists take advantage of recent revolutionary improvements in data availability and computing power to examine new hypotheses and expand our repertoire of study designs” (139). Big data may have added relevance in study designs that are patient-centric and precision-oriented (140).

“Person-oriented approaches, in contrast, focus on differences between individuals as characterized by configurations and patterns of variables. This is well in line with a precision-medicine approach to understanding disease risk, resilience, and treatment response in subpopulations of individuals” (140).

Big data is a component in studies that have shown new precision characteristics of such public health concerns as cholera (141), chikungunya (142), diabetes (143, 144), diarrhea (145), heatwave (146), influenza (147), opioid epidemic (148, 149), preterm birth (150), stunting (151), and Zika (152).

Table 1 summarizes the public health crises cited previously for which exists peer-reviewed research in at least two of the four precision public health disciplines. While the precision health research in Table 1 and in this article has peer-reviewed and exhaustive methods, there are some opportunity gaps that future research should consider and include. Table 2 lists critical gaps that occasionally exist in the research, grouped by precision public health discipline.

**Table 1:** Precision public health research leveraging big data.

	Precision public health discipline			

Public health crisis	Performing disease surveillance and signal detection	Predicting risk	Targeting treatment interventions	Understanding disease
Air pollution	(23, 24)	(101)		
Antibiotic resistance	(63)	(102)		
Diabetes		(105, 109)		(143, 144)
Diarrhea			(122)	(145)
Ebola	(83)	(106)		
HIV	(84)	(107)	(124)	
Influenza (multiple)	(71)	(103)		(147)
Malaria	(85)	(108)	(126)	
Opioid epidemic			(127)	(148, 149)
Zika		(86, 112, 113)	(129)	(152)

*Research studies (by citation) applying precision with the help of big data to a public health crisis. Public health crises are only included if big data in precision public health examples exist in more than one precision public health discipline*.

**Table 2:** Potential gaps in research methods in precision public health using big data.

	Precision public health discipline			

Study attribute	Performing disease surveillance and signal detection	Predicting risk	Targeting treatment interventions	Understanding disease
Data	Lack of clinical data, lack of attempt to build data sharing agreements to attain clinical data, or lack of attempt to use other methods to add phenotypic data about subjects No addition of traditional surveillance approach data to test incremental improvement in hybrid approaches	Lack of clinical data, lack of attempt to build data sharing agreements to attain clinical data, or lack of attempt to use other methods to add phenotypic data about subjects Novel determinants may be missed by starting with too narrow a scope Data collected in the coverage area may not be available in other areas	Molecular substrate is missing entirely, or missing within specific ethnicities or other variables Lack of showing positive treatment outcomes *via* electronic health records or detailed clinical data	Data identifying more variety or precision in disease or vector etiology is not present when such precision is available/possible Molecular substrate is missing entirely, or missing within specific ethnicities or other variables Lack of adding other variables *ex post facto* to validate homogeneity of precision subgroups

Subjects	Privacy risks not addressed; as precision increases, subjects could be uniquely identified Children not included, either by design or due to big data constraints	Children not included, either by design or due to big data constraints Lack of “*n*” in the high risk areas limits validity measure results at subject or molecular levels Lack of data collection from healthy or “healthier” subjects	Privacy risks not addressed; as precision increases, subjects could be uniquely identified Some study or disease types have low “*n*,” cannot attain high confidence levels, with no guidance for future alternatives to increase confidence levels	Lack of subject precision when such precision or finer-grain subject characterization is available/possible Some study or disease types have low “*n*,” cannot attain high confidence levels, with no guidance for future alternatives to increase confidence levels

Geography	Study was conducted in a city and no design included for applying research approaches to rural areas Limited coverage area No mention of outcomes’ ability to scale outside the study coverage area	Lack of geographical precision when such precision is available/possible Study was conducted in a city and no design included for applying research approaches to rural areas Limited coverage area No mention of outcomes’ ability to scale outside the study coverage area	Lack of plan on how to implement an intervention selectively to a high-risk geographic area or areas Lack of discussion of variability of geographic attributes that affect intervention dynamics Pilots may have been done so precisely that additional pilots in other continents or biomes need to be completed to increase validity	Lack of geographic classification included in the research or lack of geographic precision No concept of geography-as-phenotype; no epigenomic or exposomic component addressed

Scaling	Sensor, UAV or other hardware is expensive, or additional hardware is needed Study performed at a country or province level and not scalable to more precise geographies due to limitations of data availability or other factors	Machine learning approach may have been selected *a priori* rather than as a result of testing multiple methods, limiting potential to scale the approach forward No postulates for taking predictions and translating them to actions, such as prevention, intervention, programming or cures	No postulates for taking research findings and translating them to actions, such as prevention, intervention, programming or cures Study may be theoretical or not include an end-to-end pilot implementation Pilot may be missing precision disease understanding that affects long-term outcomes Lack of plan for iterative or long-term follow up	No postulates for taking research findings and translating them to actions, such as prevention, intervention, programming or cures Lack of plan to replicate disease understanding in cohorts that are more random, larger, or more homogeneous/specific

*Critical features sometimes missing from precision public health studies leveraging big data, shown by public health discipline type*.

## Contributions of Big Data

Big data offers special contributions to precision public health in enabling a wider view of health variables through linking disparate or novel data (44, 153, 154) and enabling large study populations with volumes of multiomic data to identify “molecular cohorts” (155).

The technologies behind big data make it much easier to integrate a variety of data within a study (156). For example, because big data does not require investment in an *a priori* data schema, users can bring together a variety of different data and link it when the analytics are created (157). This enables researchers to link a mélange of unstructured disease and outcome data (158, 159). In their 2017 study, Harry Hemingway, in their completion of 33 studies using linked data with a total population of two million patients, said “Our findings clearly show that research using one of the NHS greatest assets—its data—is vital to innovate improvements in disease prevention, to make earlier diagnoses and to give the best treatments” (160). The inclusion of data variety increases the number of independent variables; one novel variable—or a combination of as yet uncompared variables—could end up being significant in defining relevant precision subpopulations (161, 162).

Examples of data that has been linked to help identify more precise cohorts of populations include: longitudinal health claims data (163, 164); secondary use anonymized electronic health records (159, 165); cohort studies, health surveys, and registries (166–168); environmental variables (104); molecular data such as from the genome, exposome, microbiome, or transcriptome (169–172); “mhealth” wearable and sensor data (173); mobile phone sensing data and self-reports (174); online patient generated content (175); and the semantic web (176).

The explosion of new volumes of genomic “big data” helped make possible the precision medicine movement (177). One of precision medicine’s promises was to lead to development of new treatments for subpopulations defined by their similarities at the molecular level (178, 179). Currently, translational efforts in precision medicine often work by identifying cohorts of patients who have or lack specific genomic or molecular biomarkers (132, 180). Since today’s precision medicine works at the granularity of disease subtypes and population strata and not at the “n of one” level, contemporary precision medicine really is—when applied to community crises—an example of precision public health (2).

Researchers agree that only by using very large sample sizes will genomic studies have the proper statistical power (181, 182). “These large case–control studies are essential for boosting the statistical power needed to detect the genetic variants responsible for rare diseases and can provide the necessary knowledge for use in the clinical setting,” (183). Big data has been a necessary component in the scale-up of genomic sample sizes, enabled by the decrease in cost of gene sequencing (183). Future versions of sovereign genomics programs in over ten countries have the potential to create data sets with millions of samples (184–186). These databases should be ideal platforms for research such as genome wide association studies, which have been used with over ten thousand cases per study in public health diseases such as Alzheimer’s disease (25,000+ cases), autism (16,000 cases), high blood pressure (200,000+ cases), posttraumatic stress disorder (10,000+ cases), and smoking (50,000+ cases) (187–191).

The most sophisticated precision approaches to public health today at once include data from multiple omic disciplines, can make use of linked phenotype data, and leverage novel or recent types of computation (7, 132, 192, 193). In targeting interventions, *de novo* or improved computational methods like geospatial risk modeling, latent class modeling, social molecular pathological epidemiology, and agent-based modeling simulation all benefit from big data to better identify these “intermediate” subpopulations (49, 122, 126, 193–196).

## Risks

More work needs to be done both enumerating and evaluating the risks and challenges of using big data in precision public health.

1. Individuals could be stigmatized, even when not singularly identified, when they are stratified into small, observable cohorts, where they cannot maintain a “concealable stigmatized identity” (197).
2. Big data could enable non-consented individuals to identify patients’ or citizens’ identities either due to small cohorts or by “drilling through” the deeper and wider set of population data (198–200).
3. There are known drawbacks in increased reliance on a “high-risk” strategy, as originated by Rose, including ignoring population level determinants of health; taking focus away from a radical campaign that could have more sustainable positive effect for a larger population; risking missed interventions to borderline cases; or encouraging behaviors that continue to exist outside of social norms (201).
4. Big data risks targeting only relatively wealthier communities where data can be collected, or where big data expertise or distribution technologies are endemic (72, 202, 203).
5. For data collected through social media, crowdsourcing or similar channels, there may be more data about, in or from urban centers or areas of dense population, which will require additional computational governance (64).
6. Prevalence of large volumes of new types of individual health information available digitally risks that it could fall into the hands of unregulated commercial enterprises, or of insurance companies (204).
7. Experiencing governance gaps due to default use of existing governing legislation, rules or principles designed for data and technologies “that have now been superseded” by big data calls for more regulation (16, 205).
8. Applying novel big data without the appropriate controls, clinical interpretation, or statistical governance could lead to model overfitting, lack of accuracy, or results like Google Flu Trends, and could damage public faith in big data’s ability to add precision to public health or trust in contributing their own data (99, 206–208).
9. Big data brings unique challenges in data quality. Cai and Zhu created a big data quality framework with no less than 14 attributes by which any big data’s robustness should be assessed. Ignoring qualities like timeliness, accuracy, completeness or reliability leads to research weakness (209).
10. Performing healthcare research that includes big data is marked by, and needs, larger teams of diverse practitioners, often including informaticians, data scientists, computer scientists, physicians, researchers, and more—potentially leading to fewer studies and the challenges inherent in collaborating in large teams (59, 173).
11. Research that includes big data with high “variety” or linked data is likely to include a higher median number of data sources, which could require increased investment in cleaning and curating the data—resulting in slower scientific progress—or could compel the challenges of analyzing high dimensional data (210). For example, the high dimensionality of data found in both molecular and linked data incurs specific risk. Alyass et al. believe this data is “prone to high rates of false-positives due to chance alone…this requires researchers to adjust for multiple testing to control for type 1 error rates…or reduce dimensionality *via* sparse methods” (211).

## Conclusion

Precision public health is exciting. Today’s public health programs can achieve new levels of speed and accuracy not plausible a decade ago. Adding precision to many parts of public health engagement has led and will lead to tangible benefits. Precision can enable public health programs to maintain the same efficacy while decreasing costs, or hold costs constant while delivering better, smarter, faster, and different education, cures and interventions, saving lives.

Precision public health does not require big data. That said, the future of big data in precision public health is assured, based on its successes and acceleration of use to date. Big data and the methods created to make it useful allow precision public health practitioners to operate at the top of their license and can bring more insight to cohort membership, disease pathways and treatments. Big data enables lower costs and more precision to find, educate, track, and help each high-risk citizen. In the future, precision public health needs, imperatives, mandates and techniques will drive new capabilities into big data.

Using big data in precision public health has risks. A number of risks were identified here and future study will expand these or identify more. Protecting the dignity, privacy, security of citizens and patients, while finding truly meaningful significant outcomes in a reasonable timeframe will take effort on the part of each and every researcher in this space.

What are the calls to action? Investment has increased, but additional investment and research are needed in many areas. First, more experimentation is needed to understand how to best create and mobilize open data, open science, open source communities, and open collaboration platforms. For context, the Observational Health Data Sciences and Informatics collaborative is a thriving global open science community focused on large scale population health outcomes and prediction. If such a collaborative existed for precision public health, one imagines practitioners could leverage shared best practices, data, open software, and opportunities. Second, there are opportunity gaps in training precision public health workers in countries with a dearth of data scientists, on-premise data storage and computational assets, or access to big data. For example, communities suffering public health crises increasingly desire to “learn how to use the information and improve their ability to respond to future outbreaks in the region,” rather than having their data removed for analysis by better funded nations (212). Third, follow-on research is needed in the area of big data in precision public health. Specifically, (a) best practices in performing data quality assessment along a broad range of attributes should be enumerated, (b) existing research should be scored along these attributes as well as those studies’ compliance with statistical best practices specific to big data and high dimensionality, (c) each area of value delivery—disease surveillance, predicting risk, targeting intervention and understanding disease—needs their own full treatment with regard to methods, data sources, data management, and more, (d) some critical framework ought to be created and proposed to systematically measure precision public health studies and programs, specific to and beyond big data, and (e) as precision public health becomes more mature, emerging trends should be noticed and evaluated. Fourth, more work is needed in areas of ethics, risk, and governance. The community should be watching for overreliance on big data-driven approaches that lead to decreases in radical whole-population solutions that increase baseline health norms. Fifth, the global economic opportunity of using big data prescriptively in public health has not been systematically measured, beyond specific country or disease successes. For context, organizations such as the United Nations, the World Bank, and the United States Agency for International Development have estimated economic impacts of individual epidemics. These or other institutions could convene a task force to estimate the economic benefit of applying precision to public health responses, as well as the relative contribution of big data. Sixth, precision public health centers of excellence in universities can help. Today, leaders in schools of public health are speaking and writing about precision public health; presumably academic courses, concentrations and centers will follow in stepwise progression. Seventh, new technical innovation must continue and needs investment. For example, this could include applying deep learning to precision public health use cases, or creating a novel free and open source data science software “pipeline” for geospatial event prediction.

Future precision public health will be transformative. It will include new applications, modifications, and uses of today’s assets, including social media and communication platforms, unmanned aerial vehicles, mobile applications, mobile sequencing, self-screening, sensors, vaccine or drug internet-of-things inventions, and more. Tomorrow, we could be looking up, wondering if a high-resolution satellite is mapping our neighborhood to predict the path of an infectious disease, or if a drone is approaching with a targeted intervention. With future applications of precision public health and the speed of big data adoption, tomorrow’s new public health students and young practitioners soon won’t think of the discipline as precision public health. They will only think of it as public health.

## Author Contributions

The author confirms being the sole contributor of this work and approved it for publication.

## Conflict of Interest Statement

The author is employed by Cloudera, Inc., a provider of big data technology.
